# The Glenoid Vault Outer Cortex a new more accurate radiological reference for shoulder arthroplasty

**DOI:** 10.1051/sicotj/2021030

**Published:** 2021-05-19

**Authors:** Simon A. Hurst, Lorenzo Merlini, Ulrich Hansen, Jules Gregory, Roger Emery, Thomas Gregory

**Affiliations:** 1 Avicenne Teaching Hospital 125 rue Stalingrad Bobigny 93000 Paris France; 2 University Sorbonne-Paris-Nord, Equip Projet MOVEO, LaMSN 99 Avenue Jean Baptiste, Clement 93430 Villetaneuse France; 3 Université Paris Sorbonne Nord Campus de Bobigny, 1, rue de Chablis Bobigny 93000 Paris France; 4 Imperial College, St Mary’s Hospital Campus, Queen Elizabeth Queen Mother Building, Praed Street W2 1NY London UK; 5 Imperial College, Department of Mechanical Engineering South Kensington Campus SW7 2AZ London UK; 6 Department of Radiology, Beaujon Hospital, Paris Nord Val de Seine Hospitals, APHP 100 Avenue du General Leclerc 92110 Clichy France

**Keywords:** Shoulder, Arthroplasty, Navigation, Planning, Accuracy

## Abstract

*Introduction*: Correct positioning of the glenoid component is an important determinant of outcome in shoulder arthroplasty. We describe and assess a new radiological plane of reference for improving the accuracy of glenoid preparation prior to component implantation – the Glenoid Vault Outer Cortex (GvOC) plane. *Methods*: One hundred and five CT scans of normal scapulae were obtained. Forty six females and 59 males aged between 22 and 30 years. The accuracy of the GvOC plane was then compared against the current “gold standard” – the scapular border (SB). Measurements of glenoid inclination, version, rotation, and offset were obtained using both the GvOC and SB planes. These were then compared to actual values. *Results*: The mean difference between version obtained using the GvOC plane and the actual value was 1.8° (−2 to 5, SD 1.6) as compared to 6.7° (−2 to 17, SD 4.3) when the SB plane was used, (*p* < 0.001). The mean difference between estimates of inclination obtained using the GvOC plane and the actual were 1.9° (−4 to 6, SD 1.6) as compared to 11.2° (−4 to 25, SD 6.1) when the SB plane was used, (*p* < 0.001). *Conclusions*: The GvOC plane produced estimates of glenoid version and inclination closer to actual values with lower variance than when the SB plane was used. The GvOC may be a more accurate and reproducible radiological method for surgeons to use when defining glenoid anatomy prior to arthroplasty surgery.

## Introduction

Total shoulder arthroplasty (TSA), whether anatomic or reverse, can be a challenging procedure. Correct positioning of the glenoid component can be difficult: reasons for this include poor operative exposure, and the presence of glenoid deformity and defects [1].

Glenoid component malposition can lead to poor outcomes. This being an unsatisfactory range of motion, pain, and an increased risk of loosening – leading to early implant failure and the potential need for implant revision [2–4].

Several techniques have been suggested in the literature to address the challenge of achieving an accurate glenoid component position. These include patient-specific instrumentation (PSI), CT-based planning, navigation, and other computer-or robotic assistance. These techniques have shown reliable results in recent studies [5–8], but are often time-consuming and/or come with significant financial costs attached.

When pre-operative imaging studies are used as a foundation for operative planning having an accurate radiological reference for other measurements to be interpreted or derived from is crucial. The orientation of the scapular blade – also termed the “scapular border (SB)” – is the most commonly used such reference [9]. However, anatomical studies report significant variabilities in glenoid orientation (version, inclination, and rotation) relative to the scapula blade in normal non-arthritic scapulae [9, 10], with measures of retroversion ranging from −5° to 10° [11, 12]. All these factors influence the SB, and as a result, relying on a reference with such variability to plan or navigate in TSA may be misleading. Given the significant influence that preoperative glenoid anatomy has on the final glenoid component position [13] there is a clear need for a more reliable radiological plane for use as a reference in this area.

We therefore describe and assess a new radiological reference for determining glenoid anatomy in TSA – The Glenoid Vault Outer Cortex (GvOC). The accuracy of this reference is assessed against the SB method using a radiological methodology within a retrospective patient cohort.

The primary purpose of this work was to assess the accuracy of the new GvOC reference in obtaining values for glenoid version and inclination – these being potentially the two most influential anatomical considerations in TSA. Values for offset and rotation were also obtained. Secondarily these values were compared against those obtained using the SB plane to provide greater context.

## Methods

### Study design and setting

We performed a retrospective analysis of CT imaging of scapulae obtained from a series of total body CT scans performed between 2009 and 2017 within a cohort of patients. These were then assessed according to the inclusion and exclusion criteria detailed below.

#### Inclusion criteria

CT imaging must show at least one scapula in full, with CT sectional slices of <3 mm available, to allow for subsequent accurate 3D reconstruction. Patients aged between 20 and 30 years. The purpose of this specified age range was to overcome any bony morphologic changes due to age.

#### Exclusion criteria

The presence of significant pre-existing trauma, or any other kind of mechanical or physiological insult which may lead to distortion of the scapula bony anatomy. This did not include osteoarthritis.

After application of inclusion and exclusion criteria to the cohort 3D reconstructions of 105 scapulae were created from 57 different patients (33 males and 24 females), aged 22–30 years old. Forty-eight CT scans showed fully both scapulae, and 9 with only one scapula visualized in its entirety. The resulting 105 scapulae all underwent the same analysis using a standardised protocol performed by a single observer.

### Protocol utilised for the radiological analysis of included scapulae

Each CT scan contained between 200 and 300 DICOM images. Image processing software OsiriX MD software (Pixmeo, Geneva, Switzerland) was used to generate 3D reconstructions of each of the 105 scapulae. Region of interest (ROI) points was then positioned. The reference plane of the scapula or the SB was determined by positioning these ROI points along the near-linear lateral border of the scapula as well as along the deepest part of the near-linear supraspinatus fossa line. These two sets of points effectively formed two lines and were subsequently used to determine the SB.

**Figure 1:**
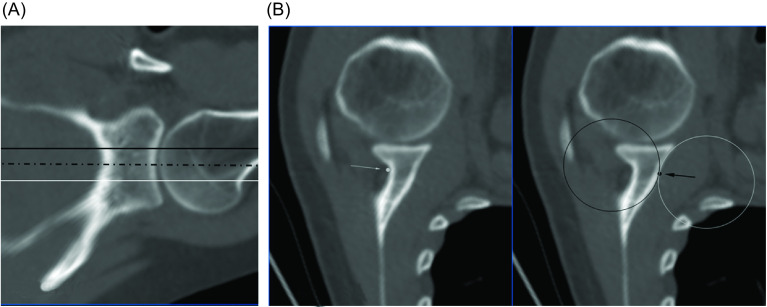
(A) CT imaging showing the position of the three axial cross-sections used to determine the GvOC ROI landmarks; *black line*; showing the axial cross-section at the level of the superior third-middle third junction; *white line*; showing the junction between the middle-third and inferior third; *dotted line;* showing the equatorial level of the glenoid. (B) CT imaging showing axial cross-sections of a glenoid specimen analysed with the GvOC ROI landmarks demonstrated. The left image; posterior ROI point – *white arrow, white dot*. The right image; The anterior slope of the glenoid is slightly curved with an anterior concavity and therefore fits with a sphere as demonstrated by the *white sphere*; and the onset of the subscapularis fossa also has a curved shape, with a posterior concavity, that also fits with a sphere as demonstrated by the *black sphere*. The anterior ROI point – *black point, black arrow* – is placed at the cross-section between both spheres, that also represents the change of curvature of the anterior aspect of the glenoid vault.

The reference plane for the GvOC was determined using three axial cross-sections strictly perpendicular to the glenoid vault: one axial cross-section at the level of the superior third-middle third junction, one at the middle-third, and the inferior third junction, and one at the equatorial level of the glenoid (Figure 1A). On each of the 3 axial views, two ROI points were placed; one at the anterior aspect and one other at the posterior aspect of the glenoid – forming a total of six points (three posterior and three anterior). The posterior ROI points were placed at the deepest part of the suprascapular nerve fossa (i.e., at the bottom of the posterior slope) and the anterior ROI points were placed at the change of curvature between the slope of the glenoid and the onset of the subscapularis fossa.

The anterior slope of the glenoid is slightly curved with an anterior concavity and therefore fits with a sphere; and the onset of the subscapularis fossa also has a curved shape, with a posterior concavity, that also fits with a sphere. This anatomical relationship allowed the anterior ROI points to be accurately placed at the cross-section between both spheres that represented this change of curvature (Figure 1B).

**Figure 2:**
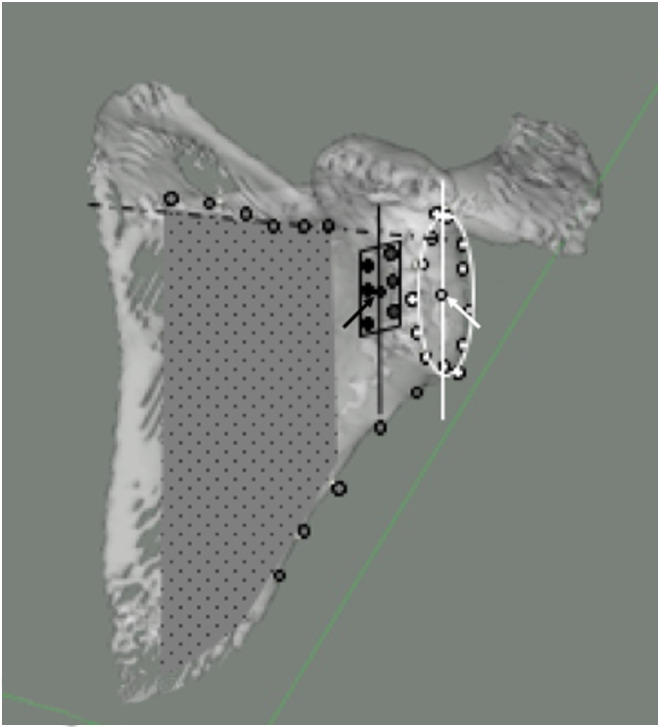
3D reconstruction of a scapula specimen showing the various radiological reference planes. GR plane (represented by the oval shape with white border) of points placed at the edge of the articular surface (White ROI points with black contour), GR centre (white arrow), and GR superior-inferior axis (white line). B plane (Grey doted area) formed by points (Grey ROI points with black contour) placed on the spine root pf the scapula (dotted line) and on the lateral border of the scapula. New GvOC plane (represented by the black rectangle) formed by points (Black ROI points) on the six described GvOC ROI landmarks, with best fit centre (Black arrow) and superior-inferior direction (Black line).

The six described GvOC landmarks formed a rectangular polygon, with a centre and a superior-inferior direction (Figure 2).

The GvOC reference plane was then defined as the best fit line which passed through all the 6 ROI points.

The other relevant bony radiological landmarks of the glenoid rim (GR), were then obtained using a previously established protocol set out by Gregory et al. [13] (Figure 2).

In order to obtain values for glenoid version and inclination independent of the SB and GvOC reference planes.

3D files containing the ROI were transferred to the 3D Reshaper mathematical software (Technodigit, Neyron, France) allowing for the relevant measurements to be determined.

Intra- and inter-observer reproducibility tests were performed on the interpretation of the results in order to assess the effect of bias. To facilitate the calculation of intra-observer reproducibility for each scapula, one observer carried out 10 repeated measures of each relative position parameter under investigation.

In a similar manner, inter-observer reproducibility was evaluated. Ten different observers assessed the same relative position parameters for each scapula. For both intra- and inter reproducibility tests, 95% confidence interval (95% CI) was calculated, using Microsoft Excel software (Microsoft, Redmond, Washington, USA).

To evaluate the significance of any difference observed between values obtained using the GvOC plane and SB plane relative to the “actual values”, the Student’s *t*-tests were performed. Probability values were calculated using a significance threshold of 0.05.

## Results

The mean difference between estimates of version using the GvOC plane and the actual was 1.8° (−2 to 5, SD 1.6) as compared to 6.7° (−2 to 17, SD 4.3) when the SB plane was used, (*p* < 0.001).

The mean difference between estimates of inclination based on the GvOC plane and the actual were 1.9° (−4 to 6, SD 1.6) as compared to 11.2° (−4 to 25, SD 6.1) when the SB plane was used, (*p* < 0.001). An overview of results for all the parameters measured for both the GvOC and the SB is shown in Table 1. Intra-observer reproducibility results to account for bias are presented in Table 2. Results showed statistically consistent results for every type of measurement undertaken in individual scapulae (*p* < 0.01). Inter-observer reproducibility results are presented in Table 3 and showed statistically reliable measures between each observer.

**Table 1:** Relative positions of SB vs. GR and GvOC vs. GR in the 105 scapulae.

Measurement	Mean	SD	Minimum	Maximum	Student *t*-test (comparison between GvOC and SB values)
Retroversion (°)					*p* < 0.001
SB/GR	6.7	4.3	2	17	
GvOC/GR	1.8	1.6	−2	5	
Superior inclination (°)					*p* < 0.001
SB/GR	11.2	6.1	−4	25	
GvOC/GR	1.9	1.6	−4	6	
Rotation (°)					*p* < 0.001
SB/GR	6.1	2.8	0	15	
GvOC/GR	1.8	1.6	−2	10	
Offset distance (mm)					*p* < 0.001
SB/GR	3.8	1.2	1.4	8	
GvOC/GR	0.3	0.3	0	1.6	

**Table 2:** Intra-observer reproducibility tests for SB/GR GvOC/GR planes position calculations in one scapula.

Measurement	Mean	SD	Minimum	Maximum	95% CI
Retroversion (°)					
SB/GR	1.1	0.6	0	2	0.001
GvOC/GR	0.8	0.6	0	2	0.001
Superior inclination (°)					
SB/GR	0.9	0.7	0	2	0.001
GvOC/GR	0.5	0.8	−1	2	0.001
Rotation (°)					
SB/GR	10.9	1.0	9	12	0.002
GvOC/GR	3.2	0.6	2	4	0.001
Offset distance (mm)					
SB/GR	2.0	0.1	1.8	2.2	0.08
GvOC/GR	0.3	0.1	0.2	0.5	0.05

**Table 3:** Inter-observer reproducibility tests for SB/GR and GvOC/GR planes position calculations in one scapula.

Measurement	Mean	SD	Minimum	Maximum	95% CI
Retroversion (°)					
SB/GR	1.3	0.9	0	3	0.002
GvOC/GR	0.8	0.6	0	2	0.001
Superior inclination (°)					
SB/GR	1.1	1	0	3	0.002
GvOC/GR	0.6	0.9	0	3	0.002
Rotation (°)					
SB/GR	11.5	1.1	10	13	0.002
GvOC/GR	3.6	1.2	2	6	0.002
Offset distance (mm)					
SB/GR	2.1	0.15	1.9	2.4	0.09
GvOC/GR	0.3	0.1	0.2	0.5	0.05

## Discussion

We describe in this paper a new radiological plane of reference the GvOC. This plane was able to reliably and consistently be found and was more accurate when compared to the traditional SB plane currently used commonly to define glenoid anatomy in TSA. The GvOC may therefore be an alternative radiological plane for surgeons to use in order to define glenoid anatomy more accurately.

This study is limited by the single-observer protocol utilised, however, this was controlled to some extent by reproducibility testing. These showed good inter-observer and intra-observer reproducibility. Another limitation is the use of a young population for analysis. The concern, therefore, exists about the applicability to an older population. We believe that these concerns need to be balanced against the need for optimal anatomy with which to compare the GvOC and SB reference planes. The objective for normal anatomy was likely achieved with reported values of glenoid rim orientation with respect to the scapula blade corresponding well to previously published data for normal patients [9, 11, 14].

We are also concerned about the effect that age could have on the relationship between GvOC and GR. Although it is worth noting this appears to have likewise not been taken into consideration in any glenoid preparation guiding system in the literature to date.

We believe that the GvOC represents an important milestone in the evaluation and understanding of existing glenoid anatomy in TSA. A key stage during TSA is glenoid preparation and implantation. This demands an understanding of the patient’s unique glenoid morphology alongside a sound surgical technique. The difficulty of achieving this is well documented in the literature [1, 7, 9, 13]. When this is not achieved it often results in a poorly positioned glenoid component and the literature reports associated poor outcomes for patients [2]. Walch et al. for example report a 32% rate of definite radiographic loosening after TSA for primary osteoarthritis [4]. Optimal bony fixation of the glenoid implant appears to be directly correlated to better radiological and clinical results. To achieve this glenoid implant placement in TSA should likely target the centre of the glenoid vault – thereby aiming for the area of maximal bone stock [1, 13]. Achieving this, however, remains a challenge [1, 15, 16].

These difficulties have contributed to the emergence of technologies now in widespread use, such as CT scan-based planning, multiplanar and 3D planning, patient-specific instrumentation (PSI), along with navigation and robot assistance [6, 17–21].

These techniques may have shown encouraging results in the literature [6, 8, 17, 19, 22], but many present a common significant limitation: high variability of the ROI bony landmarks used to predict the pre-eroded position of glenoid. This is a limitation as for example the SB plane (or the Friedman plane) as described in the radiological protocol of our study relies on a series of ROI landmarks that have high variability, and therefore can lead to a resulting plane of reference that is misleading.

The glenoid vault itself has been studied as a potentially more reliable alternative landmark for deriving the glenoid version [14, 23, 24], as well as being the optimal fixation site for the glenoid implant itself [1, 15, 25]. However, determining the glenoid vault from the complex inner cortex geometry is challenging [25]. Thus, the planning of the implant position is often based on the SB plane. This is often then further adjusted manually so that the implant fixation fits with the glenoid vault inner cortex (i.e., the maximal bone axis). This might explain some recently published data suggesting inaccurate results when using CT scan-based planning, alongside multiplanar and 3D planning [20].

There was therefore a clear need for a more accurate radiological reference plane. We believed that would be done principally by having more reliable ROI landmarks. This was therefore the basis of our work, and from which we discovered, and developed the GvOC plane. This new GvOC plane subsequently examined in this study appears to show increased accuracy when compared to the SB. We believe that this is primarily because it is constructed using a firmer foundation built on a greater number of more accurate ROI landmarks.

The data from our study is supported in the wider literature. Rispoli et al. has published results [26]: in 20 consecutive computed tomography scans obtained preoperatively in patients with primary osteoarthritis. The glenoid centre point was chosen on the glenoid surface and then projected back into the glenoid vault along the scapular axis and perpendicular to glenoid inclination. They reported that the difference from the projection of the glenoid surface centre point to the centre point at a 1.5-cm depth into the glenoid vault in the anteroposterior direction (i.e., what we defined as the offset distance) was 1.7 mm. In our study, the difference was 2 mm. In addition, they realised that the rotational axis of the glenoid rim matches with the axis of the vault although no data were given. In our studies, we report a mean rotation between GR and GvOC of 1.8° (±2°) However, Rispoli et al. analysed eroded glenoids and therefore were not able to determine the correspondence between the vault and pre-eroded surface layer inclination or retroversion.

## Conclusions

The new GvOC radiological reference plane may provide a more accurate and reproducible method for surgeons to use when defining native glenoid anatomy in TSA. Improved knowledge of the existing anatomy provides a unique opportunity for improved glenoid component position and thereby outcomes for patients. The next stage of research should focus on the stability and evolution of the GvOC reference plane in the aging patient’s scapulae.

## Conflict of interest

TG certifies that he has no financial conflict of interest (e.g., consultancies, stock ownership, equity interest, patient/licensing arrangements, etc.) in connection with this article.

SH certifies that he has no financial conflict of interest (e.g., consultancies, stock ownership, equity interest, patient/licensing arrangements, etc.) in connection with this article.

LM certifies that he has no financial conflict of interest (e.g., consultancies, stock ownership, equity interest, patient/licensing arrangements, etc.) in connection with this article.

UH certifies that he has no financial conflict of interest (e.g., consultancies, stock ownership, equity interest, patient/licensing arrangements, etc.) in connection with this article.

JG certifies that he has no financial conflict of interest (e.g., consultancies, stock ownership, equity interest, patient/licensing arrangements, etc.) in connection with this article.

RE certifies that he has no financial conflict of interest (e.g., consultancies, stock ownership, equity interest, patient/licensing arrangements, etc.) in connection with this article.
